# Limiting Onion Fly (*Delia antiqua*) and Onion Thrips (*Thrips tabaci*) Damage to Onions by Combined Use of Environmentally Acceptable Control Methods: Fact or Fantasy?

**DOI:** 10.3390/insects16111097

**Published:** 2025-10-27

**Authors:** Tanja Bohinc, Stanislav Trdan

**Affiliations:** Department of Agronomy, Biotechnical Faculty, University of Ljubljana, SI-1000 Ljubljana, Slovenia; stanislav.trdan@bf.uni-lj.si

**Keywords:** synergism, biological control, chemical control, entomopathogens, sticky boards, white clover

## Abstract

**Simple Summary:**

A two-year field experiment was conducted in order to test synergy between biological and biotechnical control measures. In this regard, we tested the individual and combined use of entomopathogenic fungi and nematodes, colored sticky boards and lures, and white clover as an intercrop for the control of onion fly (*Delia antiqua*) and onion thrips (*Thrips tabaci*) on onions. Growing onion with an intercrop of white clover was the most effective method for reducing damage from onion thrips and crop losses caused by onion maggots. However, this came with a significant drawback. The white clover was found to compete too heavily with onions. The results of our research confirm limited effectiveness of the individual or combined use of other environmentally acceptable methods of controlling the two most important insect species on onions in Central and Southern Europe.

**Abstract:**

In a two-year study (2023–2024), field experiments were conducted at a location where onion fly (*Delia antiqua* [Meigen]) and onion thrips (*Thrips tabaci* Lindeman) are permanent pests. The objective of the study was to investigate independent and combined application (synergistic effect) of environmentally acceptable methods (biological and biotechnical control methods) of controlling these pests on onions. Seven treatments were tested, including a positive control (chemical control) and a negative control (untreated plots), as well as various combinations of environmentally friendly approaches, such as entomopathogenic fungi and nematodes, white clover intercropping, and colored sticky boards with lures. The onion fly caused greater damage and subsequent bulb loss than the onion thrips in both years, despite white clover (as independent application) as an intercrop, and the combined application of white clover and entomopathogens proved to be most effective against thrips and fly damage, ultimately resulting in a suboptimal onion yield of less than 7 tons per hectare annually. It is evident that environmentally acceptable control methods were experiencing challenges in either reducing the extent of damage caused by both pests or increasing the yield of healthy bulbs. It is unfortunate that, despite observing an increased yield of healthy bulbs in certain treatments, a significant quantity of bulbs was also lost due to onion fly.

## 1. Introduction

In Slovenia, we began to intensively study the environmentally acceptable plant protection methods, together with biological control methods almost 30 years ago [1,2,3]. It is becoming increasingly important to implement sustainable pest management strategies for onion production, particularly in the fight against onion thrips (*Thrips tabaci* Lindeman) [4] and onion fly (*Delia antiqua* [Meigen]) [5]. The effectiveness of Slovenia’s registered onion pest treatments is limited, as contact insecticides for onion thrips are susceptible to rain, and the diamide insecticide for the onion fly is restricted to one application per season [6].

Onion thrips [2,4] and the onion fly [7] are among the most important pests of onion (*Allium cepa* L., Alliaceae) worldwide. Onion thrips are harmful to plants both directly and indirectly. In the first case, the appearance of silver spots on the leaves, which are a result of sucking by larvae and adults, reduces the photosynthetic activity of plants and shortens the growing season, while the cause of the second type of harmfulness of the onion thrips is the transmission of tospoviruses [8]. On the other hand, onion fly larvae can destroy up to 28 young onion plants over their developmental period [7]. Feeding on developing bulbs results in bulb distortion and creates entryways for fungal and bacterial pathogens that can cause bulb rot [9,10]. As with other insect species, the number of generations of the onion fly is determined by climatic conditions [7,11], and in Central Europe it develops three generations per year [12], with the first and second generations being the most damaging.

Alternative methods for onion thrips control have been extensively studied and described in the past. For example, the use of white clover (*Trifolium repens* L.) as an intercrop is considered one of the most effective methods of reducing onion thrips damage to onions [3], and no negative effects on the yield were found in such a cropping system. Intercropping is a system in which a plant species (the intercrop) is grown specifically to reduce pest insect damage on the main crop (onion in our research) [2]. Bird’s-foot trefoil (*Lotus corniculatus* L.) and garden savory (*Satureja hortensis* L.) have been shown in a related study to be suitable intercrops in leek cultivation [13]. The use of intercropping in onion cultivation highlights both positive and negative aspects of the yield. Negative effects on onion yield were found in the combination with pepper, while carrot as an intercrop was shown to have a positive effect on onion yield [14].

The entomopathogenic fungi *Beauveria bassiana* (Bals.-Criv.) Vuill. and *Metarhizium anisopliae* (Metschn.) Sorokin have also been employed to control onion thrips [15]. While light blue sticky boards are effective for monitoring onion thrips population levels, they fail to reduce the population of this polyphagous pest [2,3,16]. For onion fly management, alternative methods show varying results [17]: exclusion nets may negatively impact yield, whereas the use of flower belts has been shown to increase yield by up to 25% while simultaneously enhancing natural enemy presence. Entomopathogenic nematodes offer a key biological control method, proving effective against onion fly when used with insecticidal seed treatment [18] and controlling soil-dwelling stages of onion thrips [19], though they are ineffective against thrips life stages found on foliage [20].

The low efficiency of independent, environmentally acceptable methods, exemplified by poor biological control of onion thrips in Canada [17] and onion fly resistance to spinosad seed treatment in North America [21], necessitates the study of simultaneous, combined approaches [22,23]. Recent findings, such as the greater efficiency of combining spinosad with an onion fly attractant [5], demonstrate that these integrated control methods are a crucial future direction for pest management.

The aim of this study was to evaluate whether combining biological and biotechnical control methods could produce a greater reduction in damage caused by onion fly and onion thrips than either method alone.

## 2. Materials and Methods

### 2.1. Location and Period of the Experiments

The two-year experiment was conducted in 2023 and 2024 at the Laboratory Field of the Biotechnical Faculty in Ljubljana (46°04′ N, 14°31′ E, 299 m a.s.l.). In both years, ‘Sturon’ onion bulbs (supplier: KGZ Sloga, Ltd., Kranj, Slovenia) were planted with 0.20 x 0.25 cm spacing on 12 April 2023 and 15 April 2024. The total experimental area was 300 m^2^ (with 10 m^2^ plots) in the first year and 600 m^2^ (20 m^2^ per plot) in the second year.

Before planting, the soil was tilled twice to a depth of 20 cm and fertilized with NPK (7–20–30; 500 kg/ha). An additional 3 kg/10 m^2^ of chicken manure (supplier: Jata Emona, Ltd., Ljubljana, Slovenia) was incorporated in 2023. Weed control was performed manually. Yield was collected and sorted in both years on 3 August 2023 and 6 August 2024. Healthy bulbs were subsequently dried, with final post-drying sorting occurring on 21 August 2023 and 22 August 2024.

### 2.2. Experimental Layout and Spraying Intervals

The experimental design consisted of a three-block experiment in both years, with seven treatments randomly assigned per block. Treatments 1, 2, 3, 6, and 7 were covered with black polyethylene (PE) mulch supplier: Predikat, Ltd., Zdole, Slovenia). Treatments 1 and 2 served as positive control (treated with Slovenia-registered insecticides against onion fly and onion thrips) and negative control (untreated), respectively. Treatment 3 involved simultaneous evening spraying (typically on a rainy day) with a backpack sprayer (type: 425 CLASSIC, supplier: Eurogarden, Ltd., Ljubljana, Slovenia) at 1 MPa and flow rate 1.42–1.46 L/min) using an injector nozzle to apply a suspension of the entomopathogenic fungus *Beauveria bassiana* (Naturalis, 27 mL per 10 l H_2_O; supplier: Karsia, Ltd., Ljubljana, Slovenia) and entomopathogenic nematode *Steinernema feltiae* (Nemopak SF, 50 mio per 10 l H_2_O, Picount, Ltd., Mošnje, Slovenia). A similar fungal/nematode suspension was applied in Treatment 7 in combination with the sticky boards and attractants used in Treatment 6. The dates on which agrotechnical measures after planting and the spraying were conducted are presented in Table 1. In the fourth and fifth treatments, the onion bulbs were planted within an intercrop of white clover, variety ‘Apolo’, supplied by Semenarna Ljubljana Ltd., Ljubljana, Slovenia. White clover was sown as mixed intercropping. In the fifth treatment, the onions were sprayed at 7–10 day intervals with a suspension of entomopathogenic fungi and entomopathogenic nematodes, as presented in the description of Treatment 3. In the sixth treatment, white sticky boards were employed to capture adult onion flies (product: Impact Board White [20 × 24.5 cm]; supplier: Russel IPM, Deeside, Flintshire, UK), and light blue sticky boards were used to capture adult onion thrips (product: Impact Blue Glue Board [20 × 24.5 cm]; supplier: Russel IPM, UK). Both sticky board types were located directly above the canopy, in the center of the plot. Three white and three light-blue sticky boards were used per plot. We adjusted the position of the sticky boards to the height of the plants during the growing season.

In the experiment white sticky boards were used in combination with an attractant (lure) for catching onion fly adults (active substance: 2-phenylethanol and n-valeric acid; product: Seedcorn/Onion Maggot Trap; supplier: AgBio, Westminster, CO, USA), and light blue sticky boards were used in combination with an attractant (combination of two natural floral scents) for catching onion thrips adults (product: Thripnok, supplier: Russel IPM, UK). Prior to utilization, the sticky boards were bisected, thereby ensuring that the dimensions of the boards employed in the experiment corresponded to 10 × 24.5 cm. The attractants for onion fly were changed at 6-week intervals, while the sticky boards were changed at 10–14 day intervals.

**Figure 1:**
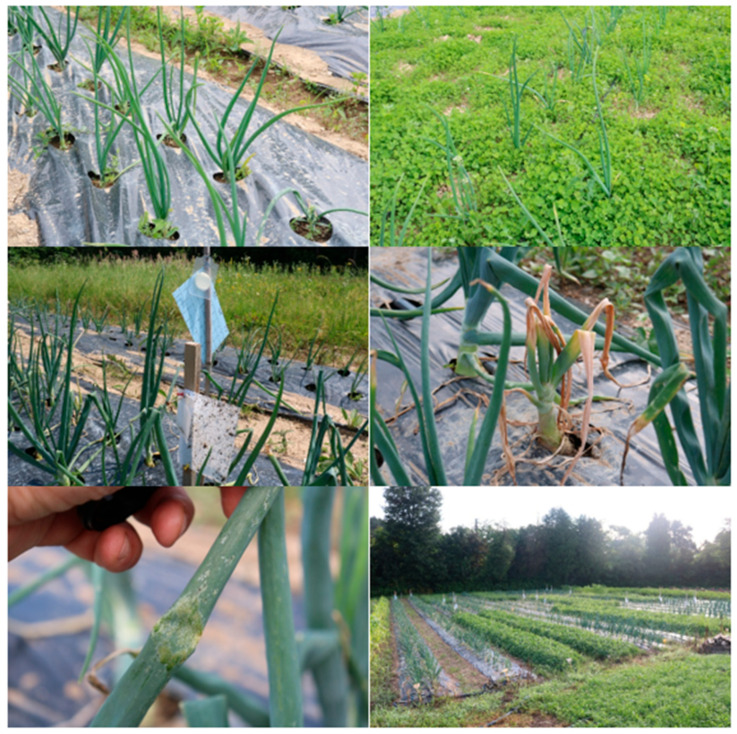
This figure shows examples of field treatments, including onions on polyethylene mulch, onion intercropped with white clover, and colored sticky traps for insect monitoring.

For foliar applications of biological control agents and phytopharmaceuticals, the wetting agent Wetcit (alcohol ethoxylate, 8.15% *w*/*w*) was added to the sprayer to improve adhesion of the preparations to the waxy onion leaves. For specific treatment in one block, one attractant and three sticky boards for specific insect species were placed. The experimental layout is also presented in Figure 1. To continue, in five out of seven treatments, onion was grown on PE mulch; in two treatments, white clover was sown as an intercrop. In two treatments white and light-blue sticky boards were used as the plant protection methods. During the growing season, we also monitored damage caused by onion thrips feeding and rotten bulbs, caused by onion fly feeding.

### 2.3. The Assessment of Onion Thrips and Onion Fly Damage, in Addition to the Monitoring of Adults on Colored Sticky Boards

**Figure 2:**
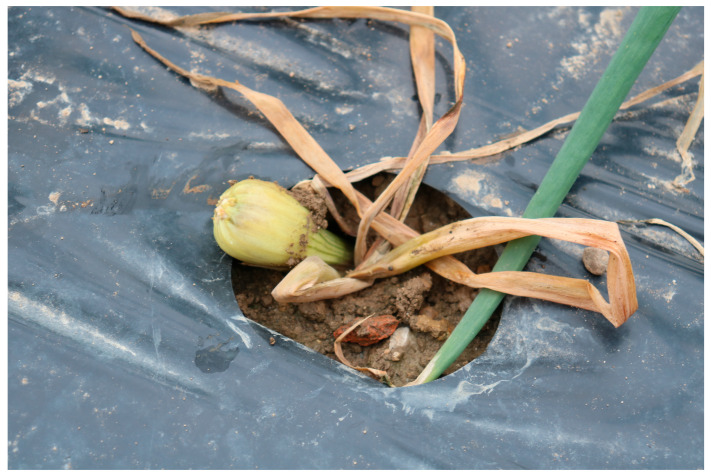
Deceased onion bulb on 13 June 2024 due to onion maggot feeding.

The impact of onion thrips (larvae and adults) on leaf damage was assessed on five plants per treatment within each block. Damage was quantified using the 6-point visual EPPO scale [24], ranging from index 1 (undamaged) to index 6 (more than 25% leaf area damaged). This method was consistently chosen over simply counting thrips numbers because the intensity of feeding and, consequently, actual harm is heavily influenced by external factors like weather (e.g., rain) and natural enemies [1,2,3,13]. Assessments were conducted three times (26 June, 3 July, 20 July) in the first year and five times (20 June, 3 July, 10 July, 18 July, 26 July) in the second year. Damage caused by onion fly larvae was monitored by counting and removing damaged bulbs on four separate occasions in both years (2023: 9 June, 21 June, 3 July, 20 July; 2024: 13 June, 1 July, 10July, 18 July). Each removed bulb was visually inspected for holes caused by maggots and then cut open to confirm secondary infection by soil fungi and bacteria. Notably, dead onion plants removed on 13 June 2024 (Figure 2) showed fly damage before any thrips feeding damage was detected. For both pest assessments, five randomly selected plants were used for thrips damage, but for onion fly damage and yield data, only bulbs in the interior of the plot were considered, excluding the outer edge.

The sticky boards were inspected and replaced at 10–14 day intervals, while the onion fly attractants were replaced monthly and the onion thrips attractants were replaced every 6 weeks. Adult individuals of both insect species were counted on both sides of the sticky boards under a stereomicroscope.

### 2.4. The Weighing of Onion Yields

The onion yield (i.e., onion bulbs and dried above-ground parts—leaves; the latter represented a negligible part of the total mass) was weighed twice: firstly, immediately in the field, and secondly, after drying. In the field, a quantitative and qualitative analysis was conducted on the plants present in each plot, with the distinction between healthy and deceased (rotten) bulbs being made. Each rotten onion that was removed was carefully examined for the presence of holes (visible to the eye) caused by onion maggots. The bulbs were then cut open to confirm subsequent infection with soil fungi and bacteria. The healthy onions were then subjected to a drying process that lasted between 14 and 21 days. This was carried out in plastic potato bags within the shaded part of the machine shed of the Department of Agronomy of the Biotechnical Faculty in Ljubljana. Following the drying process, a re-examination of the onions was conducted, resulting in the separation of the samples into two distinct categories: those deemed to be in a healthy state and those identified as being in a state of rot (rotten bulbs) due to feeding by onion maggots and then infection by soil bacteria and fungi (latent infection in the field).

We classified firm and consequently healthy onions into the group of healthy bulbs. On the other hand, we cut open every onion that was even slightly soft to check for infection by soil pathogens.

During the growing season and when sorting the crop in the field, we also counted the number of bulbs in each category (healthy/rotten). We also obtained data on the number of bulbs in each category (healthy/rotten) after drying the crop. The text provides data on the number of rotten bulbs during the growing season and during sorting in the field for each year of the experiment.

### 2.5. Weather Information

The mean monthly temperature values and monthly precipitation data were obtained for the period April–August for the both years of the experiment [25].

The average monthly temperatures ranged from 10.2 °C to 21.8 °C in the first year of the experiment, while in the second year the average monthly temperature in July and August was 24.4 °C. Precipitation was highest in the first year with almost 299 mm of precipitation measured in August 2023. In terms of temperature, 2023 was at the long-term average, while 2024 was above average from May to August. In both years there was more rainfall during the onion growing season than the long-term average for 2010–2022. In 2023, there was 66% more rainfall than the long-term average, and in 2024, there was 20% more.

### 2.6. Statistical Analysis of Results

The extent of damage on the onion leaves caused by onion thrips feeding, number of rotten bulbs caused by onion maggot feeding and yield parameters, and number of onion thrips/onion fly per sticky board were compared between different treatments. The analysis of variance (ANOVA) was conducted to establish the differences between the mean (healthy/rotten bulbs) yields per plot. Before analysis, each variable was tested for homogeneity of variances. If variances were nonhomogeneous, data were transformed to log (Y) before ANOVA. The Student–Newman–Keuls multiple range test (*p* < 0.05) was used to separate mean differences among the parameters in all treatments. Statgraphics Centurion XVI was used [26]. Data are presented as untransformed means ± SE. Detailed statistical values can be found in Table S1, under Supplementary Files.

## 3. Results

### 3.1. Damage to Onion Leaves Caused by Feeding of Onion Thrips in 2023 and 2024

We found that the average index of damage caused by onion thrips varies depending on the year of the experiment and treatment. Based on the aforementioned analysis, we also found a significant effect of interactions between the treatment and the year of the experiment.

**Figure 3:**
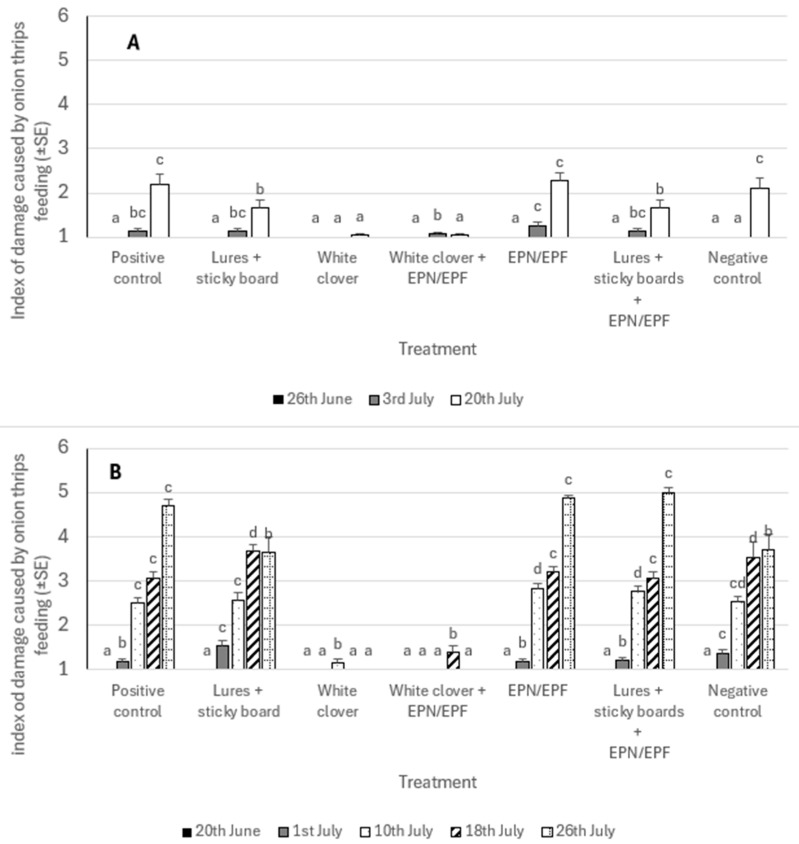
Indices of damage caused by onion thrips feeding during growing seasons in 2023 (**A**) and in 2024 (**B**). The lower case letters present differences within date of evaluation and between different treatments.

In 2023, the average index of damage to onion leaves was significantly influenced by treatment and evaluation date. Based on a general statistical analysis, we also confirmed the influence of interactions between the assessment date and the treatment. According to the general statistics, the extent of damage did not exceed an index of 2 in any of the treatments. In the first assessment period, we did not find any damage caused by onion thrips, while in the second and third periods, we found the lowest extent of damage to plants where we used white clover as an intercrop. On 20 July, we found damage levels higher than index 2 in three (positive and negative controls, application of entomopathogenic nematodes and fungi) of the seven treatments (Figure 3A).

In 2024, both the treatment and the time of assessment significantly influenced the damage index, with a notable interaction between them. The first damage was detected on 1 July, peaking in the negative control and the sticky boards/attractant combination, though it did not exceed index 2. In contrast, the treatment combining entomopathogenic fungi and nematodes recorded an initial index 1 but increased to an average of index 5 (11–25% leaf area damage) by the final assessment. The intercrop treatment with white clover showed the least damage, with virtually no impact except for a minor extent of 1.15 ± 0.08 recorded on 10 July (Figure 3B).

**Figure 4:**
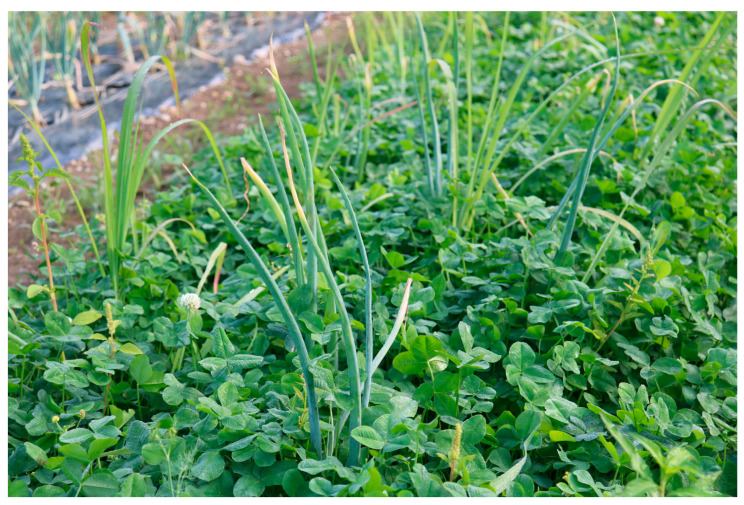
White clover at the beginning of flowering, before onion thrips feeding injuries were detected (1 July 2024).

When white clover was at the beginning of flowering (as demonstrated in Figure 4) in 2024 (July 1), the first injuries caused by onion thrips feeding were detected.

### 3.2. Effect of Onion Maggots on Bulb Decay

**Figure 5:**
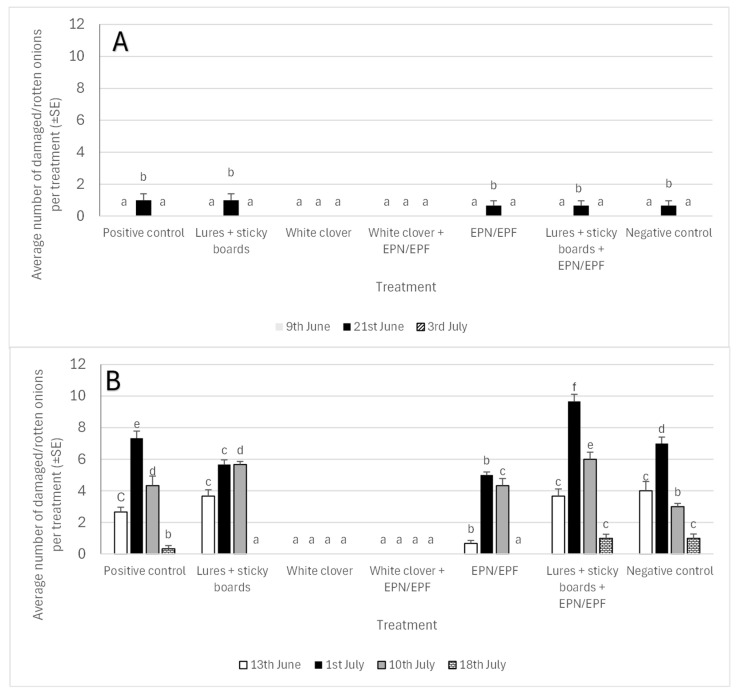
Average number of deceased onions per treatment in 2023 (part **A**) and 2024 (part **B**) due to onion fly maggots feeding (average cumulative number of damaged bulbs showing rot over the current sampling material (time since last sampling). The lower case letters present differences between treatments within specific date of evaluation.

General statistical analysis showed that the mean number of rotten bulbs varied significantly by both the year and the treatment, though their interaction was not significant. In the first year specifically, the number of bulbs decayed by onion maggots was significantly impacted by the treatment and the sampling date, but not their interaction. Rotten bulbs were observed in all treatments by 21 June, with five out of seven treatments averaging one rotten bulb each. Crucially, no rotten bulbs were found in the treatment using white clover as an intercrop (Figure 5A).

In 2023, a total of 10 bulbs were removed during the growing season (all on June 21), distributed across four treatments: 3 in the positive control, 2 in the negative control, 2 in the EPN/EPF treatment, and 3 in the lures + sticky boards + EPN/EPF treatment. During the initial yield sorting, the highest numbers of rotten bulbs were found in the lures + sticky boards (41), the negative control (38), and the positive control (35), while the lowest numbers were observed in the EPN/EPF treatment (12). The treatments involving white clover as an intercrop yielded 27 rotten bulbs, and the clover sprayed with EPN/EPF yielded 30. The combination of lures + sticky boards + EPN/EPF also resulted in 30 rotten bulbs.

In the second year of the study, the number of bulbs decayed by onion maggots differed significantly based on both the sampling date and the treatment, though their interaction was not substantial. Consistent with the first year, no bulb loss was observed in treatments where white clover was used as an intercrop. On 1 July, the highest number of rotten bulbs was recorded across all treatments, with plots using the combination of sticky traps, attractants, and the EPN/EPF suspension showing the highest average of over eight rotten bulbs during this period (see Figure 5B).

In the 2024 growing season, a total of 214 rotten bulbs were removed: the highest number came from the lures + sticky boards + EPN/EPF combination (61), followed by the negative control (45), lures + sticky boards (45), the positive control (33), and the EPN/EPF spray treatment (30). During the final field crop sorting, the highest number of rotten bulbs was found in the EPN/EPF spray treatment (105), followed by the negative control (83), positive control (82), and lures + sticky board treatment (77). The lowest numbers were recorded in the treatments involving white clover intercrop (50 for clover alone and 55 for clover with EPN/EPF), while the combined lures + sticky boards + EPN/EPF treatment yielded 62 rotten bulbs.

### 3.3. Average Number of Onion Thrips Adults on Light Blue Sticky Boards (in Treatments 6 and 7)

With the use of general statistical analysis, we found out that the treatment had no significant effect on the number of onion thrips adults on sticky boards. The year of the study had a significant effect on the number of catches (while the interactions between the two factors were not significant.

**Figure 6:**
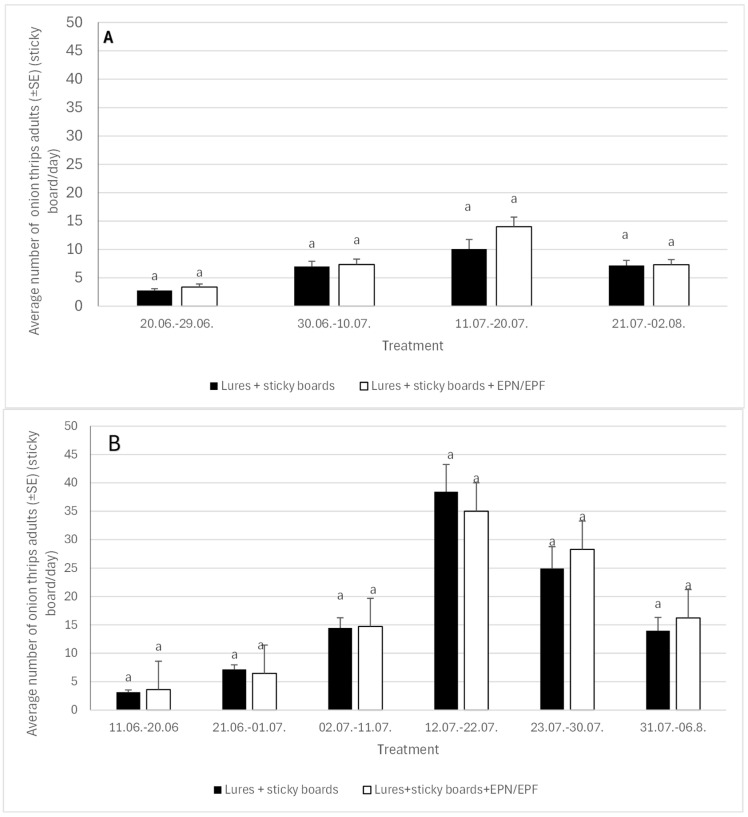
Average number of onion thrips adults (light blue sticky board/day) at different intervals in 2023 (part **A**) and 2024 (part **B**). Lower case letters present differences between treatments within time interval.

Sticky boards were replaced four times in 2023 (29 June, 10 July, 20 July, 2 August) and six times in 2024 (20 June, 1 July, 11 July, 22 July, 30 July, 6 August). The 2023 statistical analysis revealed that the assessment date significantly influenced the number of onion thrips adults captured on the light blue sticky boards, but neither the treatment nor the interaction between the factors was statistically significant. The maximum catch of onion thrips adults occurred between 11 July and 20 July, with over 10 adults per day recorded on a sticky board (see Figure 6A).

In 2024, the time interval significantly affected the number of adult onion thrips captured on sticky boards, but no significant differences were found between treatments. Consistent with 2023, the highest numbers of thrips adults were recorded in July, with a peak rate of more than 35 individuals per sticky trap per day between 12 July and 22 July, followed by a rate exceeding 25 individuals per day between 23 July 23 and 30 July 30 (Figure 6B).

### 3.4. Average Number of Onion Fly Adults on White Sticky Boards (in Treatments 6 and 7)

We can conclude that the year of investigation influenced the number of catches, while we did not find any differences in catches based on treatment.

**Figure 7:**
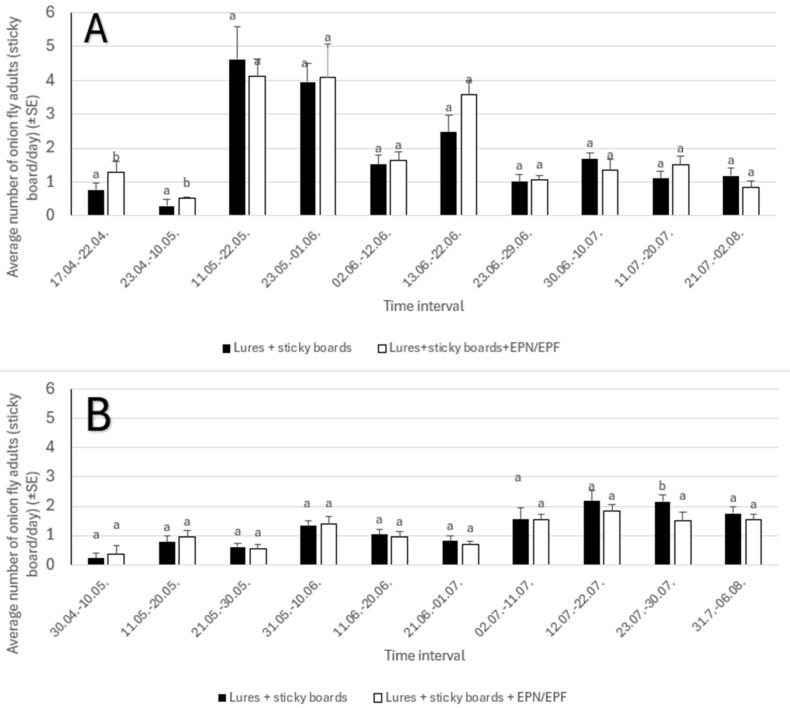
Average number of onion fly adults on white sticky boards in 2023 (part **A**) and 2024 (part **B**). The lower case letters present differences between treatments within time interval.

We found no significant differences in the average number of adult onion flies captured between treatments in either the first or second year (Figure 7A,B). However, the time interval of trap setting significantly affected the average daily catch in both years. The overall number of flies caught was higher in the first year, peaking at more than four individuals/board/day between 11 May and 22 May. In the second year, the highest catch was recorded later, between 12 July and 22 July, with an average of two individuals per sticky board per day.

### 3.5. Average (Total) Yield in 2023 in 2024

The year of evaluation and the treatment exerted a significant influence on the average total yield in the field. Furthermore, a substantial effect on yield was observed for interactions between the year of assessment and treatment. In both years, the highest yields were observed in the treatment that was sprayed with a combination of EPN/EPF, with yields exceeding 10 t/ha in both years. When white clover with or without other environmentally friendly control methods was used, the lowest yields (less than 7 t/ha) were recorded in both years.

Based on the general analysis of variance, the mean total field yield exhibited substantial variation between different treatments in the first year of the experiment, with the mean total yield recorded as 13t/ha in the positive control and 12.72 t/ha in the negative control.

**Figure 8:**
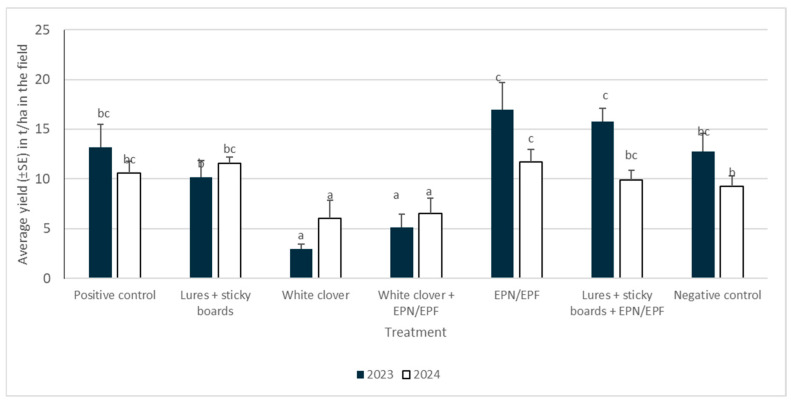
Average yield of onion bulbs in the field in 2023 and 2024 (lower case letters present differences between treatments within specific year).

The yield obtained in the treatment where EPN/EPF was sprayed onto the onions was almost 17 t (16.97 ± 0.96 t/ha). In the second year, the average total yield in the field differed depending on the treatment. In the treatments where onions were grown within white clover, the average total yield was approximately 6.5 t/ha. In the positive control, 10.6 t/ha was recorded, while in the treatment involving a combination of sticky traps and attractants, 11.67 t/ha was recorded (Figure 8).

The overall healthy and rotten yields varied significantly between treatments and years. In 2023, the highest healthy yields were recorded in the EPN/EPF spray and the lures + sticky boards + EPN/EPF combination, with both substantially exceeding the controls. Conversely, treatments utilizing white clover intercrop (alone and with EPN/EPF) consistently produced the lowest healthy yield and the highest rotten yield. The lowest rotten yield was seen in the combination treatment (lures + sticky boards + EPN/EPF).

In 2024, overall healthy yields were generally lower than the previous year. The sticky boards + lures treatment performed best, and several control and combined treatments also maintained high healthy yields. Similar to 2023, the white clover treatments again resulted in the lowest healthy crop. Rotten yield was generally reduced compared to 2023, with the lowest amount recorded in the combined clover + EPN/EPF treatment (Table 2).

### 3.6. Percentage of Healthy/Rotten Bulbs of Onion in the Field

During the experimental period, we calculated % of healthy and % of rotten bulbs in the field. Rotten bulbs were the result of secondary infections of soil pathogens, due to damage caused by onion maggots. In 2023, the percentage of healthy yield in the field was highest (more than 90%) in plots where onions were grown with an intercrop of white clover. The low percentage (less than 70%) of healthy crops in the field is a salient feature of the negative control, the treatment where we used a suspension of EPN and EPF, and the treatment where we sprayed white clover with a suspension of EPN and EPF.

In 2024, the treatment involving the spraying of white clover with a suspension of EPN and EPF is also noteworthy due to the high percentage of healthy yield (79%). Furthermore, the utilization of a combination of sticky boards with attractants resulted in a healthy yield of almost 70%.

**Figure 9:**
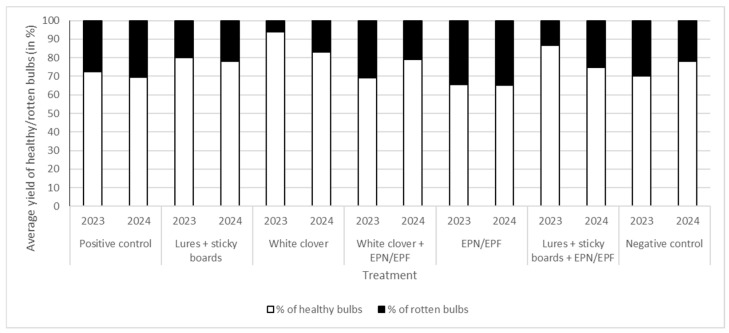
Average % of rotten and healthy bulbs of onion in the field regarding total yield in 2023 and 2024.

In the positive control, the percentage of healthy bulbs in both years was almost 70% (Figure 9).

### 3.7. Average Yield of Healthy Bulbs After Drying in 2023 and 2024

The impact of the treatments on the quantity of healthy bulbs was substantiated in both 2023 and 2024. Subsequent to the drying process in 2023, a second weighing of the yield was conducted, resulting in a measurement of over 6 t/ha of healthy bulbs in the positive control and almost 7.5 t/ha of healthy yield in two treatments. The latter involved the implementation of a multifaceted strategy that entailed the employment of sticky traps in conjunction with lures, in addition to a combination of sticky boards with lures, complemented by the incorporation of EPN/EPF suspension.

**Figure 10:**
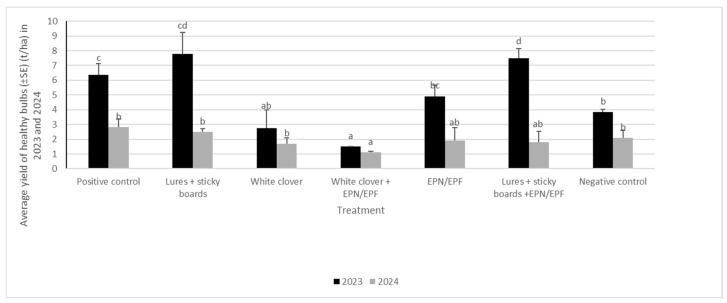
Average yield of healthy bulbs after drying in 2023 and 2024 (in t/ha) (lower case letters present differences between treatments within specific year).

In 2024, a significant variation in the weight of healthy bulbs after drying was observed among the various treatments. The lowest yield was recorded in the experiment involving the utilization of white clover as an intercrop in conjunction with entomopathogenic fungi and entomopathogenic nematodes (Figure 10).

### 3.8. Percentage of Healthy/Rotten Bulbs of Onion After Drying

**Figure 11:**
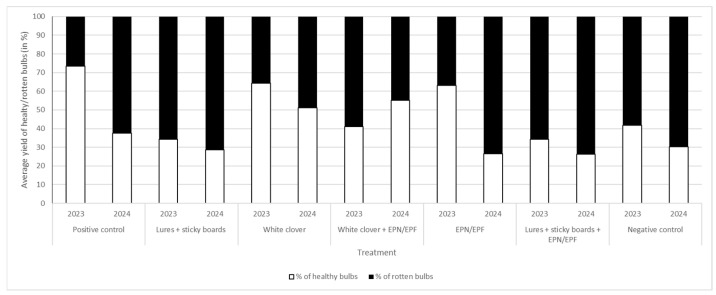
Average yield of rotten/healthy bulbs (in %) after drying in 2023 and 2024. [Rotten bulbs are the result of onion maggot feeding].

After drying, the quality of the bulbs was re-evaluated, with rotten bulbs being confirmed as the result of pre-existing latent infections stemming from onion fly maggot feeding in the field. In 2023, the positive control recorded the highest percentage of healthy yield (over 70%). The lowest healthy percentage (only 30%) was found in the sticky boards with the lure treatment, while the EPN/EPF suspension treatment yielded over 60% healthy bulbs. Notwithstanding the lower total yield in the white clover treatments in 2024, these plots exhibited the best yield quality, with over 50% of the bulbs recorded as healthy after drying (see Figure 11).

## 4. Discussion

This study showed that environmentally friendly methods, such as intercropping with white clover and using biological control agents, only offered limited protection against the onion fly and onion thrips under high pressure.

The initial damage caused by the onion thrips was observed on onion leaves in early July, which is consistent with the findings of previous studies conducted in Slovenia [2,3], while our data on the first appearance of rotten onions in mid-June due to an attack by the onion maggot is a novelty in the field of agricultural entomology in this part of Europe.

Under conditions of high pest pressure, environmentally friendly strategies—such as intercropping with white clover and the application of biological control agents—offered only limited efficacy against the onion fly and onion thrips.

In a two-year field study, various environmentally acceptable methods of controlling the two most important pests of onions in Central and Southern Europe were investigated. In view of the frequently inadequate efficacy of such methods of plant pest control when used as a single plant protection measure, a decision was taken to investigate whether the combined use of two or more environmentally acceptable methods of pest control would ensure enhanced effectiveness. In all seven treatments, the index of damage to onion leaves caused by onion thrips feeding was assessed, as was the number of onion bulbs decayed due to onion maggots feeding during the growing season.

The two-year field trial was conducted under variable weather conditions; 2024 had higher than average temperatures, while 2023 temperatures were near the long-term average. Both years experienced higher-than-average rainfall, particularly 2023. The greater damage caused by onion thrips adults in 2024 is attributed to the higher temperatures and less abundant rainfall observed that year, as hot, dry conditions are known to increase thrips populations and damage severity, while heavy rains wash thrips from plants [16].

Although white clover intercropping reduced onion thrips damage, it has also suppressed onion growth due to strong competition for light and nutrients [3].

However, the yield in this study was significantly lower in both years. Intercropping has been shown to reduce the final yield, yet concomitantly to reduce pest damage to cabbage by 48% [27]. In the second year of the study, the sowing density of white clover was reduced in comparison with the first year, but competition between white clover and onion bulbs was still detected. Therefore, this did not result in a higher onion yield. Until the final spraying with a combination of entomopathogenic fungi and entomopathogenic nematodes, it was hypothesized that the treatment involving the deployment of sticky boards in conjunction with lures, in addition to the spraying of entomopathogenic fungi/nematodes, had yielded a relatively low damage index due to feeding by the onion thrips. In both years, the percentage of healthy yield in the field in the treatment mentioned was among the highest. In the present study, the onion fly was found to be a more significant pest than the onion thrips in years with higher rainfall than the long-term average. In the absence of effective measures to control the onion fly, a 100% crop loss is to be expected [5,18,28].

In the experiment where white clover was sown as an intercrop, no rotten bulbs were observed due to onion maggots during the growing season. It is likely possible that the above-ground parts of white clover hindered the ability of onion fly females to lay eggs on or in proximity to the bulbs, as previously mentioned by [29]. Consequently, the maggots in the bulbs appear less frequently or are even absent altogether [30]. In the present study, one of the numerous methods of utilizing companion crops was selected [31], namely the sowing of white clover between onion plants. A number of options are available for the sowing of companion crops, including but not limited to staggered sowing and sowing in rows. The selected variety of white clover had very intensive growth, which made it an unfavorable variety for intercropping with onions, since onion crops are known as weak competitors because of their initial slow growth rate, development of shallow roots, and a small above-ground canopy, which does not cover the soil [32].

Foliar application of entomopathogenic nematodes (EPN) and entomopathogenic fungi (EPF) proved ineffective, as evidenced by these treatments exhibiting some of the highest levels of thrips damage and the lowest proportions of healthy bulbs and yield in both years. This outcome aligns with previous findings on the ineffectiveness of independent EPN use against onion thrips [18], particularly against foliar-inhabiting life stages [20]. Although the study used *Steinernema feltiae*, one of the most effective EPN species against thrips [19], the exceedingly wet conditions of 2023 and the high pest populations in 2024 likely exceeded the capacity of these biological control agents, preventing them from serving as effective substitutes for synthetic insecticides.

The higher rainfall recorded during both years positively influenced onion fly abundance, as evidenced by increased sticky board catches and the resulting higher number of rotten bulbs during the growing season [11,16]. This climatic factor also likely contributed to the low percentage of healthy yield in the positive control in the second year; the high rainfall in May 2024 potentially compromised the performance of the applied insecticide, cyantraniliprole, despite its established efficacy against onion fly larvae [21].

Despite the presence of onion thrips being detected on sticky boards in June, the initial damage to plants was not observed until early July.

Overall, the combined use of biological and biotechnical measures showed limited potential to replace chemical control under high pest pressure. However, these strategies may contribute to integrated pest management (IPM) programs in seasons with lower pest abundance or when used in combination with other preventive measures.

## 5. Conclusions

Under the conditions of this two-year field study, none of the environmentally friendly methods tested provided sufficient control of onion fly and onion thrips. Tested methods would be much more useful in conditions with lower abundance of tested insect species. It is widely acknowledged that crop rotation is among the most efficacious agrotechnical measures [5,16] for diminishing the population of onion fly. In this respect, the present study is among the first to emphasize the synergistic importance of selected alternative methods in controlling onion flies and onion thrips on onions. Although the tested biological and biotechnical methods were found to be only moderately effective, they could still be valuable components of integrated pest management programs, particularly in cases of moderate pest infestation. Consequently, the information and knowledge gained in this study must be further investigated in such conditions.

## Figures and Tables

**Table 1:** Dataset containing the agrotechnical measures and days of spraying.

Treatment	Date	Applied Measure
In 2023		
Treatment 1 (positive control)	12 May	Benevia (a.i. cyantraniliprole (supplier: Picount, Ltd., Mošnje, Slovenia) (at 0.75 L/ha)
	9 June	Ortiva, a.i. Azoxystrobin (supplier: Syngenta Agro, Ltd., Ljubljana, Slovenia) at 10 mL/10 l H_2_0) and natural product Algo-plasmin (supplier: Metrob Ltd., Začret, Slovenia) at 40 g/10 L H_2_O
	13 June	Karate Zeon 5 CS, a.i. lambda-cyhalotrin (supplier: Syngenta Agro, Ltd., Ljubljana, Slovenia) at 1.5 mL/10 L H_2_O
	4 July	Shirlan 500 SC, a.i. fluazinam, (supplier: Certis Belchim, Ltd., Trzin, Slovenia) at 4 mL/10 L H_2_O
	10 July	Laser 240 SC, a.i.spinosad (supplier: Karsia, Ltd., Ljubljana, Slovenia) at 4.5 mL/10 L H_2_O
	12 July	Liquid fertilizer Bio Plantella Vrt (supplier: Unichem, Ltd., Vrhnika, Slovenia) at 15 mL/1 L H_2_O
	25 July	Ortiva (supplier: Syngenta Agro, Ltd., Ljubljana, Slovenia)
	26 July	Shirlan 500 SC (supplier: Certis Belchim, Ltd., Trzin, Slovenia)
Treatment 2 (negative control)	9 June	Ortiva (supplier: Syngenta Agro, Ltd., Ljubljana, Slovenia) and Algo-plasmin (supplier: Metrob Ltd., Začret, Slovenia)
	4 July	Shirlan 500 SC (supplier: Certis Belchim, Ltd., Trzin, Slovenia)
	12 July	Liquid fertilizer Bio Plantella Vrt (supplier: Unichem, Ltd., Vrhnika, Slovenia)
	25 July	Ortiva (supplier: Syngenta Agro, Ltd., Ljubljana, Slovenia)
	26 July	Applied fungicide Shirlan 500 SC (supplier: Certis Belchim, Ltd., Trzin, Slovenia)
Treatment 3	14 April	1st spraying with combination of Naturalis (27 mL/10 L H_2_O) and Nemopak SF (50 mio/10 L H_2_O)
	25 April	2nd spraying (as described on 14 April)
	12 May	3rd spraying (as described on 14 April)
	31 May	4th spraying (as described on 14 April)
	9 June	Ortiva (supplier: Syngenta Agro, Ltd., Ljubljana, Slovenia) and Algo-plasmin (supplier: Metrob, Ltd., Začret, Slovenia)
	13 June	5th spraying (as described on 14 April)
	28 June	6th spraying (as described on 14 April)
	4 July	Shirlan 500 SC (supplier: Certis Belchim, Ltd., Trzin, Slovenia)
	11 July	7th spraying (as described on 14 April)
	12 July	Liquid fertilizer Bio Plantella Vrt (supplier: Unichem, Ltd., Vrhnika, Slovenia)
	25 July	Ortiva (supplier: Syngenta Agro, Ltd., Ljubljana, Slovenia)
	26 July	Shirlan 500 SC (supplier: Certis Belchim, Ltd., Trzin, Slovenia)
Treatment 4	12 April	Sowing of white clover var. Apolo (165 g/10 m^2^) (supplier: Semenarna Ljubljana Ltd., Ljubljana, Slovenia)
	9 June	Ortiva (supplier: Syngenta Agro, Ltd., Ljubljana, Slovenia) and Algo-plasmin (supplier: Metrob, Ltd., Začret, Slovenia)
	12 July	Liquid fertilizer Bio Plantella Vrt (supplier: Unichem, Ltd., Vrhnika, Slovenija)
	25 July	Ortiva (supplier: Syngenta Agro, Ltd., Ljubljana, Slovenia)
	26 July	Shirlan 500 SC (supplier: Certis Belchim, Ltd., Trzin, Slovenia)
Treatment 5	12 April	Sowing of white clover var. Apolo (165 g/10 m^2^) (supplier: Semenarna Ljubljana, Ltd., Ljubljana, Slovenia)
	14 April	1st spraying with combination of Naturalis (27 mL/10 L H_2_O
	25 April	2nd spraying (as described on 14 April)
	12 May	3rd spraying (as described on 14 April)
	31 May	4th spraying (as described on 14 April)
	13 June	5th spraying (as described on 14 April)
	9 June	Ortiva (supplier: Syngenta Agro, Ltd., Ljubljana, Slovenia) and Algo-plasmin (supplier: Metrob Ltd., Začret, Slovenia)
	13 June	Shirlan 500 SC (supplier: Certis Belchim, Ltd., Trzin, Slovenia)
	28 June	6th spraying (as described on 14 April)
	4 July	Shirlan 500 SC (supplier: Certis Belchim, Ltd., Trzin, Slovenia)
	11 July	7th spraying (as described on 14 April)
	12 July	Liquid fertilizer Bio Plantella Vrt (supplier: Unichem, Ltd., Vrhnika, Slovenija)
	25 July	Ortiva (supplier: Syngenta Agro, Ltd., Ljubljana, Slovenia)
	26 July	Shirlan 500 SC (supplier: Certis Belchim, Ltd., Trzin, Slovenia)
Treatment 6	17 April	Deployment of white sticky boards with lures
	9 June	Ortiva (supplier: Syngenta Agro, Ltd., Ljubljana, Slovenia) and Algo-plasmin (supplier: Metrob, Ltd., Začret, Slovenia)
	20 June	Deployment of light-blue sticky boards with lures
	6 July	Shirlan 500 SC (supplier: Certis Belchim, Ltd., Trzin, Slovenia)
	12 July	Applied liquid fertilizer Bio Plantella Vrt (supplier: Unichem, Ltd., Vrhnika, Slovenija)
	25 July	Ortiva (supplier: Syngenta Agro, Ltd., Ljubljana, Slovenia)
	26 July	Shirlan 500 SC (supplier: Certis Belchim, Ltd., Trzin, Slovenia)
Treatment 7	14 April	1st spraying with combination of Naturalis (27 mL/10 L H_2_O) and Nemopak SF (50 mio/10 L H_2_O)
	17 April	Deployment of white sticky boards
	25 April	2nd spraying (as described on 14 April)
	12 May	3rd spraying (as described on 14 April)
	31 May	4th spraying (as described on 14 April)
	9 June	Ortiva (supplier: Syngenta Agro, Ltd., Ljubljana, Slovenia) and Algo-plasmin
	13 June	5th spraying (as described on 14 April)
	20 June	Deployment of light-blue sticky boards with lures
	4 June	Shirlan 500 SC (supplier: Certis Belchim, Ltd., Trzin, Slovenia)
	28 June	6th spraying (as described on 14 April)
	11 July	7th spraying (as described on 14 April)
	12 July	Liquid fertilizer Bio Plantella Vrt (supplier: Unichem, Ltd., Vrhnika, Slovenija)
	25 July	Ortiva (supplier: Syngenta Agro, Ltd., Ljubljana, Slovenia)
	26 July	Shirlan 500 SC (supplier: Certis Belchim, Ltd., Trzin, Slovenia)
In 2024		
Treatment 1	16 May	Applied insecticide Benevia (a.i. cyantraniliprole (supplier: Picount, Ltd., Mošnje, Slovenia) (at 0.75 L/ha)
	6 June	Shirlan 500 SC (supplier: Certis Belchim, Ltd., Trzin, Slovenia) and Ortiva
	19 June	Karate Zeon 5 CS (supplier: Syngenta Agro, Ltd., Ljubljana, Slovenia) Ortiva (supplier: Syngenta Agro, Ltd., Ljubljana, Slovenia) Shirlan 500 SC (supplier: Certis Belchim, Ltd., Trzin, Slovenia)
	2 July	Laser Plus (supplier: Karsia, Ltd., Ljubljana, Slovenia)
	10 July	Ortiva (supplier: Syngenta Agro, Ltd., Ljubljana, Slovenia) Shirlan 500 SC (supplier: Certis Belchim, Ltd., Trzin, Slovenia)
	18 July	Karate Zeon 5 CS (supplier: Syngenta Agro, Ltd., Ljubljana, Slovenia)
Treatment 2	6 June	Applied fungicide Shirlan 500 SC (supplier: Certis Belchim, Ltd., Trzin, Slovenia) and Ortiva (supplier: Syngenta Agro, Ltd., Ljubljana, Slovenia)
	19 June	Ortiva (supplier: Syngenta Agro, Ltd., Ljubljana, Slovenia) Shirlan 500 SC (supplier: Certis Belchim, Ltd., Trzin, Slovenia)
	10 July	Ortiva (supplier: Syngenta Agro, Ltd., Ljubljana, Slovenia) Shirlan 500 SC (supplier: Certis Belchim, Ltd., Trzin, Slovenia)
Treatment 3	3 May	1st spraying with combination of Naturalis (27 mL/10 L H_2_O) and Nemopak SF (50 mio/10 L H_2_O)
	16 May	2nd spraying with products described on 3 May
	31 May	3rd spraying with products described on 3 May
	6 June	Shirlan 500 SC (supplier: Certis Belchim, Ltd., Trzin, Slovenia) and Ortiva (supplier: Syngenta Agro, Ltd., Ljubljana, Slovenia)
	13 June	4th spraying with products described on 3 May
	19 June	Ortiva (supplier: Syngenta Agro, Ltd., Ljubljana, Slovenia) Shirlan 500 SC (supplier: Certis Belchim, Ltd., Trzin, Slovenia)
	24 June	5th spraying with products described on 3 May
	2 July	6th spraying with products described on 3 May
	10 July	Ortiva (supplier: Syngenta Agro, Ltd., Ljubljana, Slovenia) Shirlan 500 SC (supplier: Certis Belchim, Ltd., Trzin, Slovenia)
	19 July	7th spraying with products described on 3 May
Treatment 4	16 April	Sowing white clover var. Apolo (80 g/10 m^2^) (supplier: Semenarna Ljubljana, Ltd., Ljubljana, Slovenia)
	6 June	Applied fungicide Shirlan 500 SC (supplier: Certis Belchim, Ltd., Trzin, Slovenia) and Ortiva (supplier: Syngenta Agro, Ltd., Ljubljana, Slovenia)
	19 June	Ortiva (supplier: Syngenta Agro, Ltd., Ljubljana, Slovenia) Shirlan 500 SC (supplier: Certis Belchim, Ltd., Trzin, Slovenia)
	10 July	Ortiva (supplier: Syngenta Agro, Ltd., Ljubljana, Slovenia) Shirlan 500 SC (supplier: Certis Belchim, Ltd., Trzin, Slovenia)
Treatment 5	16 April	Sowing white clover var. Apolo (80 g/10 m^2^)
	3 May	1st spraying with combination of Naturalis (27 mL/10 L H_2_O) and Nemopak SF (50 mio/10 L H_2_O)
	16 May	2nd spraying with products described on 3 May
	31 May	3rd spraying with products described on 3 May
	6 June	Applied fungicide Shirlan 500 SC (supplier: Syngenta Agro, Ltd., Ljubljana, Slovenia) and Ortiva (supplier: Syngenta Agro, Ltd., Ljubljana, Slovenia)
	13 June	4th spraying with products described on 3 May
	19 June	Ortiva (supplier: Syngenta Agro, Ltd., Ljubljana, Slovenia) Shirlan 500 SC (supplier: Certis Belchim, Ltd., Trzin, Slovenia)
	24 June	5th spraying with products described on 3 May
	2 July	6th spraying with products described on 3 May
	10 July	Ortiva (supplier: Syngenta Agro, Ltd., Ljubljana, Slovenia) Shirlan 500 SC (supplier: Certis Belchim, Ltd., Trzin, Slovenia)
	19 July	7th spraying with products described on 3 May
Treatment 6	30 April	Deployment of white sticky board with lures
	6 June	Applied fungicide Shirlan 500 SC and Ortiva (supplier: Syngenta Agro, Ltd., Ljubljana, Slovenia)
	11 June	Deployment of light-blue sticky boards
	9 June	Ortiva (supplier: Syngenta Agro, Ltd., Ljubljana, Slovenia) Shirlan 500 SC (supplier: Certis Belchim, Ltd., Trzin, Slovenia)
	10 July	Ortiva (supplier: Syngenta Agro, Ltd., Ljubljana, Slovenia) Shirlan 500 SC (supplier: Certis Belchim, Ltd., Trzin, Slovenia)
Treatment 7	3 May	1st spraying with combination of Naturalis (27 mL/10 L H_2_O) and Nemopak SF (50 mio/10 L H_2_O)
	16 May	2nd spraying with products described on 3 May
	31 May	3rd spraying with products described on 3 May
	6 June	Shirlan 500 SC (supplier: Certis Belchim, Ltd., Trzin, Slovenia) and Ortiva (supplier: Syngenta Agro, Ltd., Ljubljana, Slovenia)
	11 June	Deployment of light blue sticky boards
	13 June	4th spraying with products described on 3 May
	19 June	Ortiva (supplier: Syngenta Agro, Ltd., Ljubljana, Slovenia) Shirlan 500 SC (supplier: Certis Belchim, Ltd., Trzin, Slovenia)
	24 June	5th spraying with products described on 3 May
	2 July	6th spraying with products described on 3 May
	10 July	Ortiva (supplier: Syngenta Agro, Ltd., Ljubljana, Slovenia) Shirlan 500 SC (supplier: Certis Belchim, Ltd., Trzin, Slovenia)
	19 July	7th spraying with products described on 3 May

**Table 2:** Average healthy and rotten yield in the field, during 2023 and 2024.

In 2023	Average Healthy Yield (t/ha)	Average Rotten Yield (t/ha)
Positive control	9.39 ± 3.28	3.76 ± 1.59
Lures + Sticky boards	7.79 ± 1.43	2.34 ± 0.99
White clover	2.63 ± 0.07	9.29 ± 3.2
White clover + EPN/EPF	2.57 ± 0.11	9.21 ± 3.4
EPN/EPF	11.20 ± 1.64	5.75 ± 0.92
Lures + sticky boards + EPN/EPF	13.72 ± 2.27	2.07 ± 0.34
Negative control	6.64 ± 0.74	5.78 ± 3.2
In 2024		
Positive control	7.36 ± 1.9	3.23 ± 1.02
Lures + sticky boards	9.11 ± 0.7	2.5 ±0.4
White clover	5.00 ± 0.1	1.2 ± 0.4
White clover + EPN/EPF	5.19 ± 0.2	1.38 ± 0.5
EPN/EPF	7.63 ± 1.9	3.40 ± 0.70
Lures + sticky boards + EPN/EPF	7.39 ± 1.5	2.48 ± 1.02
Negative control	7.22 ± 0.8	2.01 ± 0.6

## Data Availability

The original contributions presented in this study are included in the article/Supplementary Material. Further inquiries can be directed to the corresponding author.

## Supplementary Materials

The following supporting information can be downloaded at: https://www.mdpi.com/article/10.3390/insects16111097/s1, Table S1: Statistical data for all the presented statistical analysis.
