# Supplementary material for: Limiting Onion Fly (Delia antiqua) and Onion Thrips (Thrips tabaci) Damage to Onions by Combined Use of Environmentally Acceptable Control Methods: Fact or Fantasy?
Source: Insects. 2025 Oct 27;16(11):1097. doi: 10.3390/insects16111097 (PMC12653390; doi:10.3390/insects16111097)
Supplement: Supplementary file 1 [file insects-16-01097-s001.zip › insects-3911135-supplementary.pdf]

Table S1. Statistical data for all the presented statistical analysis

	F	P
3.1. Damage to onion leaves caused by feeding of onion thrips in 2023 and 2024		
General Factors		
Year of the experiment	$F_{1,12}$=51.87	P<0.001
Treatment	$F_{6,12}$=24.32	P<0.001
Year of experiment x treatment	$F_{6,12}$=3.79	P<0.001
Analysis in year 2023		
Treatment	$F_{6,289}$=42.84	P<0.001
Evaluation date	$F_{2,289}$=485.01	P<0.001
Assessment date x treatment	$F_{12,289}$=37.50	P<0.001
Analysis in year 2024		
Treatment	$F_{6,1015}$=136.12	P<0.001
Assessment date	$F_{4,1015}$=367.61	P<0.001
Assessment date x treatment	$F_{24,1015}$=28.65	P<0.001

Figure 3: Indices of damage caused by onion thrips feeding during growing seasons 2023 (part A; df=(6, 105) for 3rd July, F=42.15, P<0.001; for 20th July, F=30.17, P<0.001) and 2024 (part B; df=(6,310) for 1st July, F=30.12, P<0.001; for 1st July, F=41.17, P<0.001; for 10th July, F=23.47, P<0.001; for 18th July, F=44.12, P<0.001; for 26th July, F=55.25, P<0.001).

3.2. Effect of onion maggots on bulb decay

	F	P
General factors		
Year	$F_{1,12}$=51.78	P<0.001
Treatment	$F_{6,12}$=40.13	P<0.001
Treatment x year	$F_{6,12}$=33.12	P=0.092
Analysis in 2023		
Treatment	$F_{6,50}$=19.30	P<0.001
Assessment date	$F_{3,50}$=20.10	P<0.001
Assessment date x treatment	$F_{18,50}$=20.20	P=0.096
Analysis in 2024		
Assessment date	$F_{3,160}$=25.13	P<0.001
Treatment	$F_{6,160}$=35.73	P<0.001
Assessment date x treatment	$F_{18,160}$=30.15	P=0.09

Figure 5: Average number of deceased onions per treatment in 2023 (part A) and 2024 (part B) due to onion fly maggots feeding (Average cumulative number od damaged bulbs showing rots over the current sampling material (time since last sampling). The lower case letters present differences between treatments within specific date of evaluation. (for part A, df=6,50, for June 9, F=4.12, P=0.0527, for June 21, F=3.15, P<0.001, for July 3, F=2.20, P=0.0654; for part B, df=18,160, for June 13, F=15.13, P<0.001; for July 1, F=17.30, P<0.001; for July 10, F=25.13, P<0.001; for July, F=21.21, P<0.001)

3.3. Average number of onion thrips adults on light blue sticky boards (in treatments 6 and 7)

General factors		
Treatment	$F_{6,12}$=55.30	P=0.0652
Study year	$F_{1,12}$=45.13	P<0.001
Analysis in 2023		
Assessment date	$F_{3,130}$=24.14	P<0.001
Treatment	$F_{1,130}$=4.06	P=0.0936
Analysis in 2024		
Assessment date	$F_{5,140}$=40.14	P<0.001
Treatment	$F_{1,140}$=30.30	P<0.001

Figure 6: Average number of onion thrips adults (light blue sticky board/day) in different intervals in 2023 (part A) and 2024 (part B). Lower case letters present differences between treatments within time interval (for part A, df=1,10; 20.06.-29.06., F=10.15, P=0.0564; 30.6.-10.07., F=12.17, P=0.0587; 11.07.-20.07., F=3.99, P=0.0788; 21.07.-02.08., F=9.17, P=0.7725; for part B, Df=1,14; 11.06.-20.06., F=10.14, P=0.0872; 21.06.-01.07., F=9.99, P=0.0878; 02.07.-11.07.-22.07., F=8.13, P=0.0887; 12.07.-22.07., F=3.14, P=0.0598; 23.07.-30.07., F=15.10, P=0.0777; 31.07.-06.08., F=3.12, P=0.0558)

3.4. Average number of onion fly adults on white sticky boards (in treatments 6 and 7)

General factors		
Year	$F_{1,12}$=45.12	P<0.001
Treatment	$F_{6,12}$=40.12	P<0.001
Analysis in 2023		
Treatment	$F_{1,105}$=30.14,	P=0.067
Time interval	$F_{9,105}$=20.20	P<0.001
Analysis in 2024		
Treatment	$F_{1,205}$=15.15	P=0.092
Time interval	$F_{10,205}$=15.20	P<0.001

Figure 7: Average number of onion fly adults on white sticky boards in 2023 (part A) and 2024 (part B). The lower case letters present differences between treatments within time interval. (for part A, df=1, 45, 17.04.-22.04, F=5.55, P<0.001; 22.04.-10.05, F=10.30, P<0.001; 11.05.-20.05., F=15.14, P=0.0654; 23.05.-01.06, F=14.12, P=0.0788; 02.06.-12.06., F=9.88, P=0.0778; 30.06.-10.07., F=10.15, P=0.0883; 11.07.-20.07., F=12.13, P=0.0689; 21.07.-02.08., F=17.10, P=0.0893; for part B, df=1,88, 17.04.-22.04, F=9.13, P=0.0897; 22.04.-10.05, F=17.30, P=0.0887; 11.05.-20.05., F=1214, P=0.0754; 23.05.-01.06, F=17.12, P=0.0778; 02.06.-12.06., F=11.88, P=0.0778; 30.06.-10.07., F=16.12, P=0.0883; 11.07.-20.07., F=5.13, P=0.0289; 21.07.-02.08., F=11.3, P=0.0893

3.5. Average (total) yield in 2023 in 2024

General factors		
Year	$F_{1,12}$=110.30	P<0.001
Treatment	$F_{6,12}$=144.13	P<0.001
Year x treatment	$F_{6,12}$=90.63	P<0.001
Analysis in 2023		
Treatment	$F_{6,30}$=66.12	P<0.001
Analysis in 2024		
Treatment	$F_{6,30}$=60.66,	P<0.001

Figure 8: Average yield of onion bulbs in the field in 2023 and 2024 (lower case letters present differences between treatments within specific year) (for 2023, $F_{6,130}$=50.12, P<0.001; for 2024, F=6,170, P<0.001)

3.7. Average yield of healthy bulbs after drying in 2023 and 2024

	Analysis in 2023		
Treatment		$F_{6,12}$=70.13	P<0.001
	Analysis in 2024		
Treatment		$F_{6,12}$=50.13	P<0.001
